# Supplementary material for: Closing the gap towards super-long suspension bridges using computational morphogenesis
Source: Nat Commun. 2020 Jun 1;11:2735. doi: 10.1038/s41467-020-16599-6 (PMC7264174; doi:10.1038/s41467-020-16599-6)
Supplement: Supplementary file 1 — Supplementary Information [file 41467_2020_16599_MOESM1_ESM.pdf]

# Supplementary Information

Title of manuscript: *Closing the gap towards super-long suspension bridges using computational morphogenesis*

Authors: *Baandrup et al.*

# Supplementary Information Items

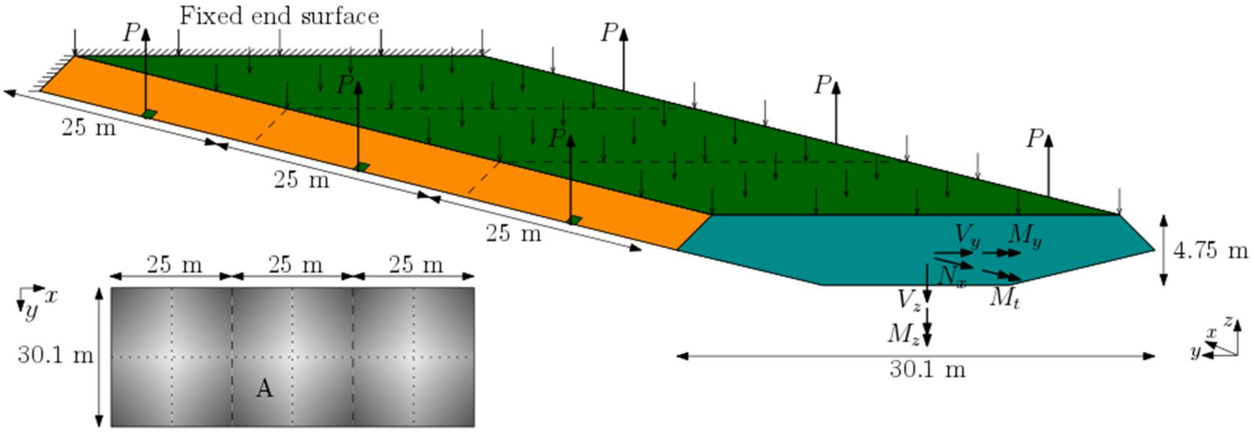


**Supplementary Figure 1 | FE model of the bridge girder.** Dimensions, loads, and boundary conditions of the three-section model of a bridge girder. Section forces were applied to the stiff end elements (blue) with $100\ \times$ modulus of elasticity and density $\rho = 1$. Distributed load was applied to the solid top elements (green) and hanger forces $P$ to the solid hanger attachments (green), both with $\rho = 1$. Design domain indicated by orange, $0 \leq \rho \leq 1$. Mapping of the active design domain A to the remaining model is indicated on the inserted illustration by graded color. Here, dashed lines indicate section borders, and dotted lines indicate symmetry lines.

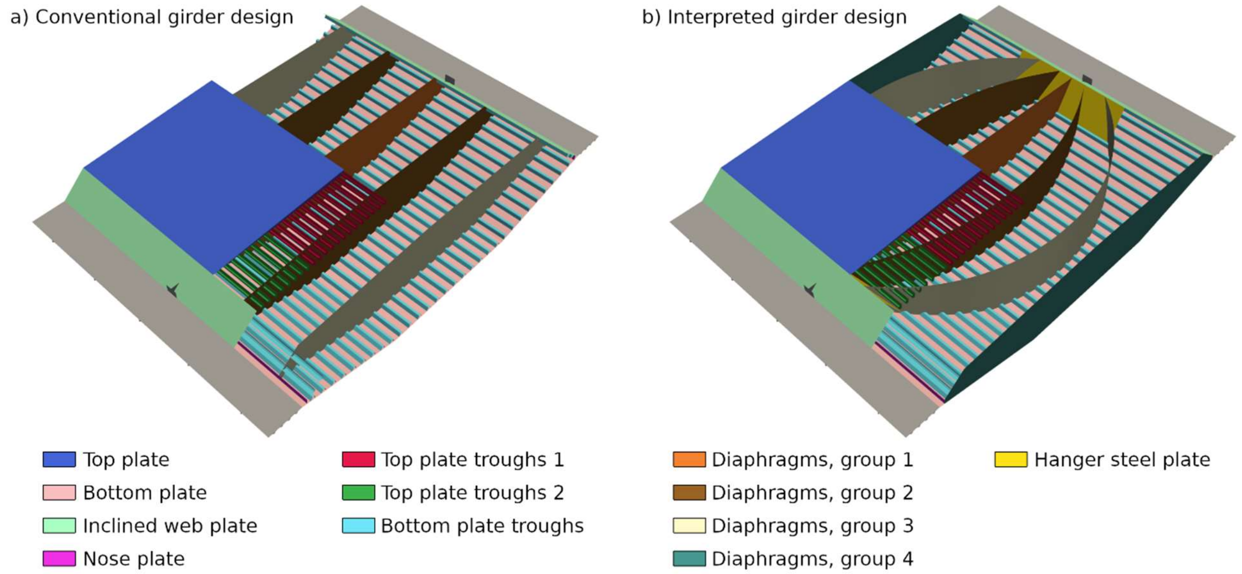


**Supplementary Figure 2 | Design variables in parametric optimization.** Indication of design variables during parametric optimization (steel plate thicknesses) on a single girder section.

**Supplementary Table 1 | Performance of the conventional and interpreted designs.** Weighted compliance of the five load cases (LC) included during the optimization and the in total 14 load cases, respectively. Furthermore, average of the maximum von Mises stresses for the five load cases is shown. Relative improvements and changes are compared to the conventional design.

| Design | Weighted compliance 5 LC [ J ] | Improvement | Weighted compliance 14 LC [ J ] | Improvement | Average of max von Mises stress [ MPa ] | Change |
|---|---|---|---|---|---|---|
| Conventional design | 264.6 | - | 655.6 | - | 225.4 | - |
| Interpreted design | 231.0 | 12.7% | 574.8 | 12.3% | 248.6 | 10.3% |
| Interpreted design - parametric optimization | 189.6 | 28.4% | 473.9 | 27.7% | 199.2 | -11.6% |

**Supplementary Table 2 | Total quantities of steel, concrete, and $CO_2$ for the entire bridge.** Estimated material quantities calculated by knock-on effects from the savings in girder self-weight. The $CO_2$ calculations only take account of material quantities, hence disregarding construction methods.

| Design | Steel [ ton ] | Savings [ ton ] | Concrete [ $m^3$ ] | Savings [ $m^3$ ] | $CO_2$ [ ton ] | Savings [ ton ] |
|---|---|---|---|---|---|---|
| Conventional design | 69,000 | - | 175,000 | - | 233,000 | - |
| Interpreted design | 63,000 | 6,000 | 162,000 | 13,000 | 213,000 | 20,000 |
| Interpreted design - parametric optimization | 56,000 | 13,000 | 146,000 | 29,000 | 190,000 | 43,000 |

**Supplementary Table 3 | Loads applied to the FE model.** Load case 1-12: global, load case 13-14: local. *Only distributed load on half of the top surface and average hanger force due to skew load.

| Load case | $N_x$ [ MN ] | $M_y$ [ MNm ] | $M_z$ [ MNm ] | $V_y$ [ MN ] | $V_z$ [ MN ] | $M_t$ [ MNm ] | Distributed load [ kN per $m^2$ ] | Hanger force [ MN ] |
|---|---|---|---|---|---|---|---|---|
| 1 | 9.3 | 65.6 | 8.5 | 0.2 | 0.6 | -23.1 | -6.8 | 2.3 |
| 2 | -16.6 | -99.6 | 52.2 | -0.1 | 0.5 | 47.8 | -12.4 | 3.7 |
| 3 | -2.1 | -247.3 | -18.7 | -0.2 | 1.4 | -26.4 | -12.5 | 3.5 |
| 4 | 4.7 | 169.3 | 6.0 | 0.0 | 0.5 | -34.3 | -7.5 | 2.3 |
| 5 | 0.1 | 28.8 | -818.2 | 4.6 | 1.1 | -10.0 | -7.8 | 2.3 |
| 6 | -1.3 | -20.8 | 403.0 | 5.4 | 0.3 | 24.4 | -8.8 | 2.5 |
| 7 | 0.1 | -13.1 | -344.2 | -5.3 | 0.3 | -7.9 | -7.8 | 2.3 |
| 8 | -1.3 | -20.8 | 403.0 | 5.4 | 0.3 | 24.4 | -8.8 | 2.5 |
| 9 | 6.1 | -36.7 | -5.9 | -0.1 | 1.3 | 55.3 | -9.2 | 3.0 |
| 10 | -13.4 | -125.1 | -25.6 | 0.0 | -0.7 | -47.4 | -11.6 | 2.5 |
| 11 | -0.1 | -13.5 | 226.5 | 0.2 | 1.0 | -170.8 | -11.4 | 3.4 |
| 12 | -2.2 | -18.4 | -235.8 | -0.2 | 1.0 | 165.5 | -11.0 | 3.2 |
| 13 | - | - | - | - | - | - | -5.0 | 1.6 |
| 14* | - | - | - | - | - | - | -5.0 | 0.8 |

**Supplementary Table 4 | Steel plate thicknesses in conventional and interpreted designs.** Steel plate thickness of the various girder parts are shown for the conventional design, and the interpreted design, before and after parametric optimization, respectively. All values are given in millimeters.

| Design | Top plate | Bottom plate | Inclined web plate | Nose plate | Top plate troughs 1 | Top plate troughs 2 |
|---|---|---|---|---|---|---|
| Conventional design | 14.0 | 9.0 | 12.0 | 10.0 | 7.0 | 8.0 |
| Interpreted design | 14.0 | 9.0 | 12.0 | 10.0 | 7.0 | 8.0 |
| Interpreted design - parametric optimization | 12.0 | 14.5 | 25.4 | 4.9 | 4.0 | 4.0 |

| Design | Bottom plate troughs | Diaphragms, group 1 | Diaphragms, group 2 | Diaphragms, group 3 | Diaphragms, group 4 | Hanger steel plate |
|---|---|---|---|---|---|---|
| Conventional design | 6.0 | 10.0 | 10.0 | 10.0 | - | - |
| Interpreted design | 6.0 | 6.9 | 6.9 | 6.9 | 6.9 | 8.3 |
| Interpreted design - parametric optimization | 4.0 | 8.3 | 6.3 | 5.9 | 6.3 | 8.8 |
