# Supplementary material for: Closing the gap towards super-long suspension bridges using computational morphogenesis
Source: Nat Commun. 2020 Jun 1;11:2735. doi: 10.1038/s41467-020-16599-6 (PMC7264174; doi:10.1038/s41467-020-16599-6)
Supplement: Supplementary file 3 — Description of Additional Supplementary Files [file 41467_2020_16599_MOESM3_ESM.docx]

Description of Additional Supplementary Files

**Title: Supplementary Software**

• Abaqus

o ConventionalDesign.cae – Abaqus model file of the conventional design

o ConventionalDesign.inp – Abaqus input file of the conventional design

o InterpretedDesign.cae – Abaqus model file of the interpreted design

o InterpretedDesign.inp – Abaqus input file of the interpreted design

o InterpretedOptDesign.cae – Abaqus model file of the interpreted and optimized design

o InterpretedOptDesign.inp – Abaqus input file of the interpreted and optimized design

• Stl

o Conventional_OneSection.stl – STL file of one section of the conventional design

o Interpreted_OneSection.stl – STL file of one section of the interpreted design
